# Astrocyte Gliotransmission in the Regulation of Systemic Metabolism

**DOI:** 10.3390/metabo11110732

**Published:** 2021-10-26

**Authors:** Cahuê De Bernardis Murat, Cristina García-Cáceres

**Affiliations:** 1 Helmholtz Diabetes Center, Helmholtz Zentrum München, German Research Center for Environmental Health (GmbH), Institute for Diabetes and Obesity, 85764 Neuherberg, Germany; cahue.bernardis@helmholtz-muenchen.de; 2 German Center for Diabetes Research (DZD), 85764 Neuherberg, Germany; 3 Medizinische Klinik and Poliklinik IV, Klinikum der Universität, Ludwig-Maximilians-Universität München, 80336 Munich, Germany

**Keywords:** astrocytes, calcium signaling, energy balance, gliotransmission, systemic metabolism

## Abstract

Normal brain function highly relies on the appropriate functioning of astrocytes. These glial cells are strategically situated between blood vessels and neurons, provide significant substrate support to neuronal demand, and are sensitive to neuronal activity and energy-related molecules. Astrocytes respond to many metabolic conditions and regulate a wide array of physiological processes, including cerebral vascular remodeling, glucose sensing, feeding, and circadian rhythms for the control of systemic metabolism and behavior-related responses. This regulation ultimately elicits counterregulatory mechanisms in order to couple whole-body energy availability with brain function. Therefore, understanding the role of astrocyte crosstalk with neighboring cells via the release of molecules, e.g., gliotransmitters, into the parenchyma in response to metabolic and neuronal cues is of fundamental relevance to elucidate the distinct roles of these glial cells in the neuroendocrine control of metabolism. Here, we review the mechanisms underlying astrocyte-released gliotransmitters that have been reported to be crucial for maintaining homeostatic regulation of systemic metabolism.

## 1. Introduction

The field of neuroscience has experienced significant advancement in knowledge on how the brain processes information as a result of the growing evidence supporting that glial cells, as with astrocytes, are fully integrated into neuronal networks, thus forming one functional regulatory circuit required for brain function [1]. In addition to serving as a support system, active functions have been assigned to astrocytes, including the control of cerebral vascular remodeling and blood flow [2], and the regulation of all aspects of neuronal function, such as neurogenesis [3], neuronal transmission [4], and synapse formation/elimination and homeostasis [5], among others. By providing energy substrates and neurotransmitter precursor molecules via the astrocyte-neuron lactate shuttle [6] and the glutamate/γ-aminobutyric acid (GABA)-glutamine cycle [7], astrocytes ensure adequate neuronal metabolism, connectivity, and brain functioning. The characteristic star-like shape of astrocytes possesses specific non-overlapping territorial domains and hence fills the local environment, interacting with a large number of synapses that can dynamically change depending on the surrounding microenvironment in response to neuronal activity and/or metabolic status for the regulation of physiological responses [1,8]. Remarkably, astrocytes play a key role in neurotransmitter clearance [1] and spatial K^+^ buffering [9], which support neurotransmission homeostasis. Astrocytes, as with neurons, sense and respond to metabolic [8] and synaptic cues [10] through specific metabolic and neurotransmitter receptors/transporters expressed along their membranes, thus influencing the active state of synapses to which they are often intimately associated [11]. Seminal findings demonstrated that astrocytes display changes in their intracellular Ca^2+^ concentration [12], a signal that appears to be the relevant signal for astrocytic responses [4,13]. As secretory cells, astrocytes possess the molecular machinery to send molecules and ions back and forth, which are essential in regulating all physiological processes (e.g., synaptic connectivity) required for a normal brain function [1,14].

### Astrocyte Gliotransmission: The Hallmark of Astrocyte Communication

**Figure 1:**
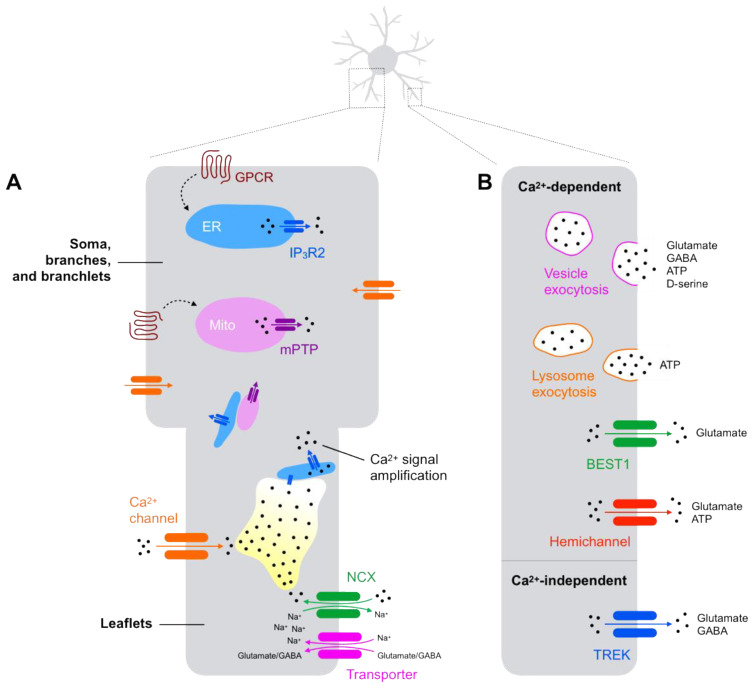
Mechanisms underlying intracellular Ca^2+^ rises and gliotransmitter release from astrocytes. (**A**) At the soma, branches and branchlets of astrocytes, the activation of G-protein-coupled receptors (GPCRs) following increased synaptic activity triggers cytosolic Ca^2+^ rises by several mechanisms, such as via the 1,4,5-trisphosphate receptor type 2 receptor (IP_3_R2)-dependent mobilization Ca^2+^ from the endoplasmic reticulum (ER) or the efflux of Ca^2+^ through mitochondrial membrane permeability transition pores (mPTPs). At fine processes of astrocytes, i.e., leaflets, increased synaptic activity may promote the co-transport of neurotransmitters and Na^+^ into the cytosol, the latter increasing the activity of Na^+^/Ca^2+^ exchangers (NCX) that results in cytosolic Ca^2+^ elevations. Additionally, Ca^2+^-permeating channels contribute to the influx of Ca^2+^ in astrocytic leaflets. The Ca^2+^ influx into leaflets may trigger local Ca^2+^ transients and propagate the Ca^2+^ signaling to distant domains via its signal amplification mediated by a Ca^2+^-dependent Ca^2+^ release from ERs via the IP_3_R2 pathway; (**B**) Several mechanisms account for the release of gliotransmitters from astrocytes. Mostly, these processes occur in a Ca^2+^-dependent manner via the exocytosis of vesicles. Lysosome exocytosis, bestrophin1 (BEST1) channels and hemichannels have also been described to participate in Ca^2+^-dependent gliotransmitter release mechanisms. Moreover, the involvement of a Ca^2+^-independent release of gliotransmitters via two-pore domain K^+^ (TREK) channels is reported.

Despite the absence of membrane electrical excitability, astrocytes exhibit a marked ionic handling in response to diverse stimuli, which is crucial for proper regulation of physiological processes controlled by the brain [1]. For instance, internal K^+^, Na^+^, Ca^2+^, and H^+^ fluctuations in astrocytes are associated with increased synaptic activity whereas Cl^-^ permeability is involved in astrocyte volume changes. Since compelling evidence suggests the existence of Ca^2+^-dependent astrocyte-neuron communication [4,13], intracellular Ca^2+^ signaling has been extensively studied in astrocytes. Several works have reported that an enhancement in synaptic activity may result in astrocyte Ca^2+^ rises following the activation of specific metabotropic G-protein coupled receptors (GPCRs) by synaptic neurotransmitter spillover, such as glutamate [15,16], GABA [17], ATP [18], acetylcholine [19,20], and dopamine [21]. Intracellular Ca^2+^ events at the soma, primary branches, and branchlets of astrocytes are greatly mediated by the inositol 1,4,5-trisphosphate receptor type 2 (IP_3_R2) signaling pathway that mobilizes Ca^2+^ from the endoplasmic reticulum to the cytosol. However, astrocyte Ca^2+^ responses may occur in an IP_3_R2-independent manner, especially at their fine processes, i.e., astrocyte leaflets, in which Ca^2+^-permeating channels [22] and Na^+^/Ca^2+^ exchangers [23] underlie the mechanism of Ca^2+^ entry into the cytosol. Ca^2+^ transients in astrocyte processes may also occur via Ca^2+^ efflux from mitochondrial membrane permeability transition pores (mPTPs) [24]. Therefore, there are multiple sources of Ca^2+^ that contribute to the increased cytosolic Ca^2+^ content in response to synaptic activity (Figure 1), highlighting the complex Ca^2+^ dynamics within astrocyte cellular compartments, ranging from slow, global Ca^2+^ events to rapid, local Ca^2+^ transients [25,26,27,28]. In turn, astrocyte Ca^2+^ elevation promotes the release of signaling molecules, such as glutamate, ATP, D-serine, and GABA, which can influence the activity of neighboring neurons and other cells to ultimately modulate local metabolism and the information processing within neuronal networks, a process known as gliotransmission [4]. Such signaling molecules are released from astrocytes through several intracellular pathways including vesicle-mediated exocytosis and diffusion through channels (Box 1). The complex intracellular Ca^2+^ dynamics in astrocytes and the great variety of mechanisms releasing their gliotransmitters suggest distinct, specific roles of astrocyte gliotransmission depending on the spatial location, quality, and intensity of the stimulus as well as the gliotransmitter releasing site and astrocyte interactions with the surrounding microenvironment (Figure 1).

Box 1 **Astrocytes release gliotransmitters via several pathways.**

*Vesicle-mediated exocytosis*
 The soluble N-ethylmaleimide-sensitive factor attachment protein receptor (SNARE)-mediated vesicular exocytosis is likely the major mechanism for the Ca^2+^-sensitive release of gliotransmitters from astrocytes. Using ex vivo brain slices from mice and human, it was observed that Ca^2+^-dependent astrocyte-released glutamate induces the activation of N-methyl-D-aspartate receptors (NMDARs) in neurons triggering slow inward currents [29,30,31,32], an effect greatly attenuated by disrupting the SNARE complex [33,34,35]. These currents have also been shown to be associated with changes in neuronal excitability and neurotransmission. Accordingly, vesicular glutamate transporters and SNARE proteins are localized in astrocyte processes adjacent to neurons [35]. The blockade of vesicular exocytosis also impairs the release of ATP from astrocytes, which may influence synaptic transmission and behavioral responses [36,37,38,39]. Likewise, the exocytosis of lysosomes is also thought to participate in ATP release from astrocytes [40,41].

*Diffusion through channels*
 In addition to exocytotic mechanisms, the release of astrocyte gliotransmitters may occur through ion channels. For instance, glutamate can be released via Ca^2+^-activated bestrophin 1 (BEST1) channels localized at astrocyte microdomains [42] to modulate synaptic plasticity [43,44]. BEST1 channels are also permeable to GABA, which may tonically inhibit neighboring neurons [45,46,47] and drive pathological mechanisms following its impaired release [48,49]. Moreover, astrocytes are able to release gliotransmitters via hemichannels [50,51,52,53,54] and Ca^2+^-independent pathways, such as two-pore domain K^+^ channels [42,55].

## 2. Physiological Processes by Which Astrocytes Regulate Systemic Metabolism

Although diverse studies have reported the mechanisms underlying Ca^2+^ responses and gliotransmitter release from astrocytes in the regulation of local metabolism and synapse physiology [1,2,4], their influence on the control of systemic metabolism has recently begun to be explored. Understanding the communication between astrocytes and neighboring cells involved in whole-body counterregulatory responses to metabolic challenges may add relevant insights on how physiological processes are controlled by the brain. In this section, we aim to describe the contribution of astrocyte gliotransmission to the modulation of the surrounding microenvironment and synaptic transmission that are related to the homeostatic regulation of systemic metabolism and behavior.

### 2.1. Cerebral Vascular Integrity and Remodeling

**Figure 2:**
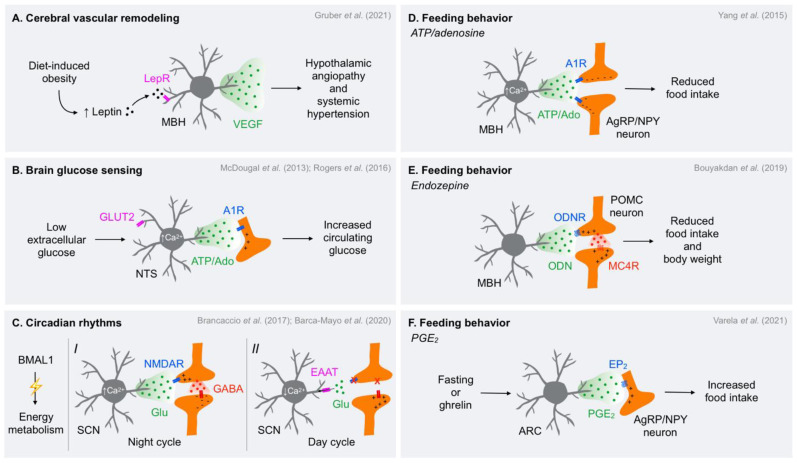
The action of astrocyte-released gliotransmitters in the control of systemic metabolism. (**A**) Diet-induced obesity promotes hyperleptinemia, which hyperactivates leptin receptors (LepRs) in astrocytes from the mediobasal hypothalamus (MBH) and leads to the release of vascular endothelial growth factors (VEGFs), promoting hypothalamic angiopathy and systemic hypertension; (**B**) Astrocytes from the brainstem nucleus tractus solitarius (NTS) sense extracellular glucose concentration drops via glucose transporter type 2 (GLUT2) and respond with ATP/adenosine (Ado) release, leading to the activation of adenosine A1 receptors (A1Rs) in neighboring neurons to restore normoglycemia; (**C**) The disruption of the brain and muscle ARNT-like protein-1 (BMAL1) signaling in astrocytes impairs energy metabolism. (I) During the night cycle, astrocytes from the suprachiasmatic nucleus (SCN) show increased Ca^2+^ transients, which induce the release of glutamate that binds to N-methyl-D-aspartate receptors (NMDARs) subtype 2C in presynaptic neurons resulting in increased γ-aminobutyric acid (GABA)-mediated neurotransmission; (II) In the day cycle, astrocytes are silent and the glutamate near the synaptic cleft is taken up by astrocytic excitatory amino acid transporters (EAATs), therefore reducing the GABAergic tone onto SNC neurons; (**D**) Ca^2+^ rises in astrocytes from the MBH promote the release of ATP/Ado that acts in presynaptic neurons and/or postsynaptic agouti-related protein/neuropeptide Y (AgRP/NPY) neurons to reduce food consumption; (**E**) Astrocytes from the MBH release the endozepine octadecaneuropeptide (ODN), which acts on its receptor in proopiomelanocortin (POMC) neurons, leading to the activation of the upstream melanocortin-4 receptor (MC4R) signaling to reduce food intake and body weight; (**F**) Fasting/ghrelin may activate astrocytes from the arcuate nucleus of the hypothalamus (ARC), promoting the release of prostaglandin E_2_ (PGE_2_) to increase the activity of AgRP/NPY neurons, ultimately inducing food intake.

The brain stores a low amount of energy [56] and largely depends on oxidative metabolism for supporting its energy requirements [57]. Therefore, a constant, adequate supply of glucose and oxygen from the brain vasculature is needed in order to match the high metabolic demand of neurotransmission and brain function [58,59,60]. In this regard, astrocytes are situated in a strategic position to control continuous fuel supply to the brain by enveloping virtually all brain blood vessels with their endfeet [61] and making close contact with synapses by their processes [11], thus regulating the cerebral vascular tone to accomplish neuronal function in both resting and active states. In the last decade, multiple studies have highlighted the active role of vascular endothelial growth factors (VEGFs) in angiogenesis and vascular architecture in the brain [62] by modulating tight-junction proteins in blood vessels for controlling blood-brain barrier (BBB) permeability [63,64,65,66]. Astrocytes have been shown to be the predominant source of VEGF within the brain [67,68], as the blockade of astrocytic VEGF-dependent releasing mechanisms attenuates BBB leakage in animal models [68,69,70]. Other studies have pointed out that angiogenesis correlates with higher astrocyte density and elevated VEGF expression levels in the brain of mice and humans [71]. Notably, additional studies have reported that a hypercaloric diet rapidly increases the number of astrocytes in the hypothalamus [72] and promotes angiogenesis and endothelial dysfunction in both rodents and humans [73,74]. Recently, it has been revealed that VEGF-derived hypothalamic astrocytes are directly involved in obesity-induced hypothalamic microvasculature remodeling and elevated systemic blood pressure via sympathetic outflow, an effect dependent on leptin signaling and concomitant with the onset of obesity [75] (Figure 2A). Further, the selective disruption of the hypoxia-inducible factor 1α-VEGF signaling cascade in astrocytes protected mice against obesity-induced hypothalamic angiopathy, increased sympathetic drive, and arterial hypertension [75]. These findings reveal the astrocyte-released gliotransmitter VEGF as a relevant molecule involved in the tuning of sympathetic outflow controlling cardiovascular function and challenge the traditional view that microvascular complications in the brain are derived from arterial hypertension [76].

### 2.2. Brain Glucose Sensing

Astrocytes are highly glycolytic cells [6] and exhibit higher glucose transport and utilization in comparison to neurons [77]. Using a fast-responsive machinery, astrocytes do not only sense extracellular glucose drops but also monitor interstitial glucose presumably to elicit autonomic responses to restore normoglycemia. Among several glucose transporters (GLUTs) expressed in astrocytes [78], GLUT1 is the predominant active isoform at the cell membrane and plays a marked role in basal glucose uptake [79]. Astrocytes also express GLUT2, which has a low affinity for glucose [80,81,82], providing a wide range of sensitivity to changes in glucose availability. Notably, GLUT2 expression in astrocytes, but not in neurons, has been reported to be necessary and sufficient to increase plasma glucagon levels in response to hypoglycemic conditions in mice [83]. The hypothalamus and the hindbrain are well-known glucose-sensing central areas [84], particularly due to their close location to brain ventricles. Here, we report evidence from the literature that describes how hypothalamic and hindbrain astrocytes may modulate local circuits and systemic metabolism in response to glucose concentration fluctuations.

#### 2.2.1. Hypothalamus

Hypothalamic neurons are capable of directly responding to changes in systemic glucose levels [85,86]. Application of glucose induces Ca^2+^ rises in tanycytes—specialized glial cells lining the floor of the third ventricle located exclusively in the mediobasal hypothalamus—which promote the release of ATP via connexin 43 (Cx43) hemichannels acting on neighboring tanycytes through purinergic P2Y1 receptor to result in cellular activation by an IP_3_R-mediated Ca^2+^ signaling [87,88]. Although astrocytic Ca^2+^ rises in response to glucose fluctuations have not been demonstrated in the hypothalamus yet, hypothalamic astrocytes are markedly involved in the regulation of glucose homeostasis [8]. Particularly, insulin signaling in hypothalamic astrocytes is essential for adequate glucose transport into the brain and systemic glucose handling [89]. Other findings have also pointed out that elevated glucose levels lead to reductions in astrocyte coverage on proopiomelanocortin (POMC) neurons—an effect associated with increased excitatory synaptic input onto these neurons [90]. Moreover, hypothalamic astrocytes induce insulin secretion in response to acute intracarotid injection of glucose [91], presumably via Cx43-containing gap-junction functioning [92].

#### 2.2.2. Hindbrain

Similar to the hypothalamus, the hindbrain is strongly involved in counterregulatory responses to hypoglycemia [84]. The nucleus tractus solitarius (NTS) is the primary central site receiving afferent glycolytic inputs from peripheral domains [93]. The NTS also contains astrocytes sensitive to extracellular glucose fluctuations [94], as is the case in neurons [95,96]. Intriguingly, glucose deprivation triggers Ca^2+^ rises in astrocytes via the phospholipase C-IP_3_ signaling pathway [97], an effect preceding the Ca^2+^ responses in neighboring neurons [94]. Recent studies have also reported that astrocyte purinergic signaling underlies counterregulatory responses to limited glucose availability via an NTS-arcuate nucleus of the hypothalamus (ARC) circuit. In particular, infusion of 2-deoxyglucose (2-DG), a non-metabolizable glucose analog that mimics hypoglycemic conditions, into the fourth ventricle induces blood glucose elevation in rats, an effect dependent on astrocyte integrity and adenosine A1 receptor (A1R) signaling [98] (Figure 2B). Moreover, functional astrocytes are required for purinergic P2 receptor-dependent activation of tyrosine hydroxylase (TH)-expressing NTS neurons in response to glucose deprivation [99]. Importantly, NTS^TH^ neurons can bidirectionally modulate the electrical activity of orexigenic agouti-related protein/neuropeptide Y (AgRP/NPY) and anorexigenic POMC-expressing neurons in the ARC to promote food intake in response to glucoprivic conditions [100]. Notwithstanding, the ability of ATP-mediated astrocyte signaling in tuning an NTS-ARC neuronal circuitry to ultimately modulate feeding behavior remains to be shown.

### 2.3. Feeding Circuits

Feeding is driven by an intricate neuronal network that encompasses homeostatic energy balance and hedonic responses [101]. External sensory information, vagal inputs, and circulating nutritional signals converge and are processed in the brain to then adjust feeding behavior according to whole-body energy demands [102]. Remarkably, the melanocortin system has been greatly studied as being the main integrator and control center of hunger circuits [103], and its dysfunction is directly linked with the development of metabolic diseases [104]. Two melanocortin neuron populations in the ARC with opposite functions play essential roles in the control of energy intake and expenditure: activation of AgRP/NPY-expressing neurons induces rapid and marked food seeking and consumption [105,106,107,108] whereas activation of POMC-expressing neurons promotes satiety and energy expenditure [105,109,110]. Notably, the postnatal genetic ablation of AgRP/NPY [111,112] or POMC neurons [112,113,114] results in starvation-induced death or obesity, respectively. A great deal of evidence supports that astrocytes are active players as regulators of these feeding responses by interacting with melanocortin neurons [89,115,116,117]. Specifically, several studies have reported that astrocytes within the mediobasal hypothalamus (MBH) are capable of responding to energy-related signals, such as hormones and nutrients, in order to modulate neuronal and behavioral responses required for maintaining whole-body energy homeostasis [8]. Indeed, the postnatal ablation of leptin receptors (LepRs) in astrocytes reduces hypothalamic astrogenesis [118] and leads to a retraction in primary processes coverage on melanocortin neurons in the ARC—the latter of which is associated with changes in neuronal excitability and alterations in feeding behavior [116]. Accordingly, astrocyte-specific LepR knockout induces astrogliosis in the hypothalamus of mice, blunts hypothalamic pSTAT3 signaling, and contributes to diet-induced obesity [119]. As with leptin, the disruption of insulin signaling in hypothalamic astrocytes also promotes metabolic alterations mainly due to a defect in brain glucose sensing, resulting in an aberrant systemic glucose handling [89]. Furthermore, the same line of studies has observed that the ingestion of high caloric meals triggers rapid astrocyte-neuron rearrangements, including astrocyte reactivity and alterations in the synaptology of melanocortin neurons [115]; most of these cellular events were observed prior to body weight gain [72], suggesting their potential role in promoting obesity.

#### 2.3.1. Identified Gliotransmitters by Which Astrocytes Regulate Feeding Behavior

##### ATP/Adenosine

Astrocytes have been reported to mediate feeding control via purinergic gliotransmission. Specifically, it was reported that mice reduce food consumption in response to chemogenetic Ca^2+^-dependent activation of MBH astrocytes, an effect associated with decreased firing activity of AgRP neurons following adenosine A1R activation [120] (Figure 2D). Accordingly, optogenetic stimulation of MBH astrocytes leads to an increase in extracellular adenosine content, preventing long-term fasting-induced food intake, which is abolished by A1R antagonist injection [121]. These results indicate that MBH astrocytes can release ATP—being converted to adenosine in the extracellular compartment—or adenosine itself [122] to promote anorexigenic effects by decreasing the activity of AgRP/NPY neurons. Nevertheless, it is not clear whether adenosine directly reduces the excitability of AgRP/NPY neurons, presumably by the opening of G-protein-coupled inwardly rectifying K^+^ channels associated with A1Rs [123,124,125], or inhibits presynaptic glutamatergic neurons via A1R activation, as observed in other brain regions [21,126,127]. On the contrary, other studies have shown opposing results using a similar approach with chemogenetic activation of astrocytes, but only those exclusively located in the ARC. In this case, the authors have observed that astrocyte activation promotes food consumption by increasing the orexigenic drive of AgRP/NPY neurons [128], although no potential gliotransmitter involved in this mechanism was reported. The divergent findings when exploring the role of astrocytes in the control of feeding behavior might reside in the intricate nature of neuronal circuits confined to the MBH requiring hypothalamic nuclei with opposing roles in the control of metabolism. Therefore, millimetric stereotaxic variations in the affected area may target distinct astrocytic-neuronal circuits involved in the diverse effects of feeding responses.

Other hypothalamic centered lines of investigation have shown that astrocytes located in the dorsomedial nucleus of the hypothalamus (DMH) are involved in the satiety effect of cholecystokinin (CCK), a well-known anorexigenic gut-derived peptide hormone, via purinergic gliotransmission [129]. Astrocytes respond to CCK through their CCK receptors (CCKRs) expressed along the membrane [130,131] via a Ca^2+^-dependent mechanism [129,131]. Specifically, CCKR type 2-dependent astrocyte activation triggers the release of ATP that in turn activates P2X receptors in inhibitory neurons, culminating in increased GABA release at the synapse level. Additionally, astrocyte mGluR5 was shown to be necessary for the CCK-mediated effects on GABAergic neurotransmission [129]. Indeed, astrocyte mGluR5 acts as a sensor of synaptic transmission and is markedly involved in astrocyte-neuron gliotransmission [15,16,31]. Overall, these findings suggest that the detection of glutamatergic activity by astrocytes at nearby synaptic clefts may modulate the release of ATP from astrocytes to fine-tune the information processing triggered by CCK signaling in the DMH.

Additional studies have shown the involvement of extra-hypothalamic astrocytes in feeding regulation. In this regard, the selective activation of astrocytes within the brainstem dorsal vagal complex (DVC) induces morphological changes in NTS astrocytes and reduces food-seeking behavior and food consumption, even following overnight fasting [132]. The latter effect was associated with increased c-Fos immunoreactivity, as subrogate marker for neuronal activation, in neurons from the DVC and lateral parabranchial nucleus but not in the paraventricular nucleus of the hypothalamus [132], suggesting that the astrocyte-mediated anorexigenic drive from the brainstem DVC may activate alternative circuitries to the melanocortin system.

##### Endozepines

The acyl-CoA-binding protein (ACBP) is a ubiquitously expressed cytosolic molecule that acts: (i) in intracellular pathways controlling lipid metabolism [133] or (ii) to generate and release regulatory peptides namely endozepines, such as ACBP itself, octadecaneuropeptide (ODN), and C-terminal octapeptide (OP) [134]. Remarkably, ACBP and ODN expression levels are enriched in the hypothalamus [135,136], particularly in glial cells [137,138,139,140]. Indeed, multiple evidence support that astroglial-released endozepines play a key role in the regulation of energy homeostasis. Particularly, it was shown that central administration of ODN or OP decreases food consumption in rodents and fish [140,141,142,143] by reducing NPY and enhancing POMC mRNA expression levels in the ARC [144]. Moreover, the hyperphagic response to central infusion of 2-DG is attenuated by co-infusion of OP [137]. In vitro studies from rodents also support that astrocytes are able to release endozepines upon stimulation [145,146]. Amongst several brain areas of action, astrocytes from the MBH were demonstrated to be required for triggering an anorexigenic effect via endozepine release [138]. A selective genetic manipulation of ACBP in astrocytes from the ARC is sufficient to modulate feeding behavior and body weight control. Interestingly, ACBP-expressing astrocytes are in close opposition with POMC neurons in the ARC [138], and ODN or OP application activates hypothalamic POMC neurons, as observed in ex vivo brain slices [138,140]. Given that ODN-induced food intake reduction is abolished in melanocortin-4 receptor (MC4R) knockout mice [138], astrocyte-released endozepines appear to drive an anorexigenic effect via the melanocortin system by modulating POMC neuron excitability and MC4R-dependent signaling transmission (Figure 2E). Likewise, it is thought that ODN binds to melanocortin neurons via an uncharacterized GPCR [142,147]. Central infusion of ODN-GPCR agonists attenuates food intake in mice and fish [138,140,142,143], which is associated with increased excitation of POMC neurons in the ARC of mice [138]. Accordingly, the central administration of an ODN-GPCR antagonist suppresses ODN-induced anorexigenic effects [138,142,143]. Emerging findings also suggest that leptin signaling in tanycytes is required for ODN-induced anti-obesogenic effects in mice [140], indicating the importance of the crosstalk between astrocytes and other glial cells for satiety control. Nevertheless, astrocyte-derived endozepine actions in feeding behavior appear not to be restricted to hypothalamic areas. Astrocytes from the brainstem area postrema and NTS within the DVC have been found to be enriched with ACBP and ODN protein levels [140,148]. Consistent with the hypothalamic centered studies, central administration of ODN or OP induces marked c-Fos immunoreactivity of NTS neurons accompanied by food intake inhibition [140], while blunting the swallowing reflex in mice [148]. Given that ACBP has also been shown to have CNS-independent effects on the promotion of appetite, energy storage, and obesity in mice [149], further investigations should be performed to disentangle the peripheral and central contributions of endozepines in whole-body energy balance.

##### Prostaglandin E_2_

A recent study has shown that fasting, ghrelin administration, or GABA-mediated AgRP neuron signaling increases astrocyte coverage and lowers the number of inhibitory inputs onto AgRP neurons in the ARC, an effect accompanied by depolarization of the membrane potential of neighboring astrocytes [150]. Additionally, the authors observed that the application of astrocyte-derived gliotransmitter prostaglandin E_2_ (PGE_2_) increases the firing activity of AgRP/NPY neurons from ex vivo brain slices whereas the blockade of PGE_2_ receptor EP_2_ abolishes ghrelin-induced food consumption [150] (Figure 2F). These findings indicate that rearrangements between surrounding astrocytes and AgRP-dependent circuits in a pre-feeding condition could facilitate the actions of the PGE_2_ in the activity of those neurons to promote feeding.

### 2.4. Circadian Rhythms

The circadian rhythm is present in virtually all cells of almost all living organisms. The cellular clock relies on oscillatory patterns of transcription factors based on a transcription-translation negative feedback loop (TTFL) mechanism. This process ensures the synchronization of biological mechanisms in an adequate time scale according to the active and resting phases [151]. The active phase is markedly characterized by high energy expenditure and nutrient consumption whereas the resting phase is associated with tissue repair, waste clearance, and memory consolidation [151,152]. Notably, the suprachiasmatic nucleus of the hypothalamus (SCN) is one of the major centers in coordinating the whole-body circadian rhythm [151], which influences feeding/fasting patterns and thus metabolic control [153]. In fact, lesions in the SCN elicit alterations in the daily pattern of circulating glucose, fatty acids, and insulin [154]. Besides the marked role of SCN neurons in the control of circadian behavior [151], astrocytes have recently emerged as important players in the regulation of neuronal circuits involved in the circadian rhythms, and in consequence, in whole-body energy metabolism. Specifically, the lack of the clock gene brain and muscle ARNT-like protein-1 (BMAL1) in astrocytes leads to increased food intake, body weight gain, impaired glucose handling, and shorter lifespan in mice [155]. Such changes are associated with alterations in the expression pattern of clock genes in SCN neurons and also affect circadian locomotor activity in mice [156,157,158]. These effects seem to be driven by the inability of astrocytes to control extracellular GABA content [155,157,159]. Considering that the vast majority of neurons in the SCN are GABAergic [160] and the synchronization of clock neurons in the SCN highly depends on GABAergic transmission [161,162], astrocytes may exert relevant modulation on the inhibitory circuitry dictating circadian oscillations via GABA homeostasis regulation. Indeed, the cooperative orchestration of the activity fluctuations of neurons and astrocytes in the SCN governing the circadian rhythm has gained new insights since the observation that neurons are active during the active phase of the circadian rhythm whereas astrocytes are active during the resting phase, as evidenced by Ca^2+^ measurements [158]. In this study, the authors also showed that Ca^2+^ variations in astrocytes match the release of glutamate, which binds to NMDAR subtype 2C in pre-synaptic GABAergic neurons and enhances the inhibitory drive onto SCN neurons to control behavioral rhythms (Figure 2C). On the other hand, GABAergic tone is reduced during the resting cycle by decreased release of glutamate and elevated glutamate clearance via excitatory amino acid transporters by astrocytes, thereby facilitating SCN neuron activity [158]. Strikingly, astrocytes can sustain their circadian molecular oscillations for many days even in culture [163]. Such oscillations in astrocytes endow autonomous cell-specific molecular patterns in vivo, which are sufficient to control circadian behavior via glutamate-mediated astrocyte gliotransmission within the SCN, regardless of the TTFL functioning in surrounding neurons [164]. Therefore, the circadian rhythm function highly relies on the tuning of GABA-mediated signaling by glutamatergic astrocyte-neuron communication in the SCN.

## 3. Concluding Remarks

Unlike neurons, showing long and static projections for delivering long-distance messages, astrocytes occupy small domains defined by their finger-like thin processes to influence local circuitries. Therefore, it is not surprising that astrocytes are very plastic cells with multiple functional roles and a high capacity to adapt their cytoarchitecture, gene profile, and activity in response to local neuronal demands. Despite occupying small territories, an astrocyte can physically interact with multiple synapses (estimated number > 100 synapses)—a fact that highlights the vast amount of neuronal information that a single astrocyte can process in a short amount of time. In recent years, notable progress has been made to elucidate many aspects of astrocyte physiology and gliotransmission by using the most advanced neurophysiological techniques. However, the individual distinctions of each astrocyte together with its intricate interactions with neuronal circuitries and the complex Ca^2+^ dynamics at different levels of its compartments have challenged researchers in the field to further understand how communication occurs between astrocytes and neighboring cells. Therefore, studies focused on how astrocytes decode external signals into spatial and temporal gliotransmitter release depending on the microdomain environment and its interactions would also be fundamental to shed more light on these paradigms.
